# Spanish Costaleros’ Physical Activity and Their Quality of Life

**DOI:** 10.3390/s20195641

**Published:** 2020-10-02

**Authors:** José Luis Ubago-Jiménez, Félix Zurita-Ortega, Pilar Puertas-Molero, Gabriel González-Valero

**Affiliations:** Department of Didactics of Musical, Plastic and Corporal Expression, University of Granada, 18071 Granada, Spain; jlubago@ugr.es (J.L.U.-J.); felixzo@ugr.es (F.Z.-O.); ggvalero@ugr.es (G.G.-V.)

**Keywords:** costaleros, adults, quality of life, physical activity, accelerometer

## Abstract

(1) Physical activity is one of the most influencing factors in people’ quality of life. Likewise, the costaleros of the Holy Week of Andalusia (Spain) carry out an important effort with high intensity during an extended time without any preparation. This study was the aim of knowing the intensity of the physical activity practiced by the costaleros in relation to their quality of life. (2) A transversal study was carried out with 1057 costaleros in Andalusia (Spain), where 930 were male and 127 female, between the ages of 18–61 years old (31.26 ± 7.60). For this purpose, descriptive, inferential, and correlative analyses were developed. Accelerometers (ActiGraph) were used during the procession to know the intensity of physical activity and the SF-36 test to know the self-perceived state of health and quality of life. (3) The intensity of physical activity practiced by costaleros is moderate, and it is related with their quality of life. In addition, positive associations are found between general health and physical activity. (4) Participants’ quality of life is associated with physical activity and freedom from injury. In addition, the measurement by accelerometry provides real data on the intensity of the effort made.

## 1. Introduction

Folk holidays are based around many traditions with a significant social, cultural, and indeed, religious component. Easter week in the Southern of Spain is a good model. Although it still maintains a religious sphere, it also has a strong cultural and artistic dimension expressed in its sculptures, processions, and how it is presented, as well as a tourism component [1].

One of the greatest touristic attractions are the “thrones” placed in streets by the different fraternities. Most of the thrones are transported by men and women who are called “costaleros (costaleros: people who bear on their own body the processions during Holy Week in Spain)“. Currently, three modalities of carrying the thrones during the Holy Week exist. The first and most popular is to carry the throne on the seventh cervical vertebra that can be placed behind the neck. Among the different types of costaleros, there are the “de costal” one because they use this clothing to protect the cervical area from friction and weight bearing.

The second most frequent way of carrying thrones is on shoulders. Its principal characteristic is to support the weight of the thrones on both shoulders. In this modality, men and women do not protect the area, but it is the throne itself are covered by padding. In spite of the previous types of carrying, there is another modality based on carrying a weight on a single shoulder. This method is less accepted by the Andalusian population, although it is in the city of Málaga where all the thrones are carried by this technique. Just like in the case of the shoulder-weight mode, the costaleros are not protected, as the thrones use the padding to protect them. Depending on the size of each throne, between 30 and 45 costaleros are usually carried. Costaleros do aerobic physical exercise during most of the time [2] during procession. Costaleros are also required to have a strong ability to coordinate their four physical capacities: strength, speed, resistance, and flexibility [3,4]. In fact, resistance is the most required skill in the costaleros’ effort when they reach 120 beats per minute, due to accumulated fatigue or to relays accomplished. In this regard, the most needed muscle groups are those of human body and legs, both agonists and antagonists, using isometric and isotonic contraction. Speed is used to react quickly when the throne is lifted, while flexibility is required to increase post-exercise recovery and strengthening of the most commonly used muscle groups during the effort [5]. The correct coordination between these skills is essential to be able to carry weight between 30 and 50 kg in an explosive and anaerobic manner [6]. Moreover, costaleros are not professionals and, therefore, to practice this activity is a hobby for everyone.

In order to understand the kind of physical activity practiced by a costalero, a comparative analysis is made with power lifting [7,8,9]. The aim of weightlifting is to lift a maximum weight in a one-time repeat [10,11], whereas the costalero lifts a lower weight for a longer period of time. Both practices are similar to squatting. In weightlifting, the squat is performed by putting a weight bar over the upper back and shoulder, flexing hips/knees until thighs are parallel to the floor, and then pressing weight back to the initial vertical position [12,13]. A very similar position to be performed by the costalero each time they lift up the throne. On the other hand, the weightlifting is done without movement, while the costalero once lifts a weight and has to walk with it.

Considering processions as a physical activity where an optimal physical condition is required, it is also necessary to prepare costaleros with specific workouts. Trainings are only held during a month and a half before a procession period and once a week, costaleros do procession simulation training. These sessions are shortened, although they are more intensive than the procession in general. These trainings have a duration of between one and two hours, while the processions have an average duration of around eight hours. In contrast, the weight of the throne in the training sessions is greater than in the procession to balance out the duration. Considering all the above, it can be affirmed the costaleros do vigorous physical activity at several periods during the procession. Most of it is done with moderate physical activity.

Any physical activity brings a risk for some kind of injury if the person has not been trained regularly [14,15]. Whatever the training a person has done, a small risk of injury is always present. However, according to a study carried out by the Costalero Care Centre [16], where more than 900 men and women were attended to during Easter Week, users’ main injuries were: abrasion and blisters caused by friction or pressure caused by the weight they were carrying. Cervical pain, dorsal pain, lumbagos, paraesthesia of the arms, hypertonia, muscular strain in twins, and hamstrings reduction. In addition, the main muscle groups with the highest incidence of injury are the supraspinous, scalene, scapular elevators, trapezium, lumbar, and quadriceps.

Therefore, it is necessary to know the level of training as well as physical activity intensity during the procession. Consequently, the aims of this investigation are (1) to know the intensity of the physical activity practiced by the costaleros in relation to their quality of life; (2) to establish relationships between quality-of-life conditions and injuries suffered by costaleros.

## 2. Materials and Methods

### 2.1. Subjects and Design

The research has followed a transversal, cross-sectional, and non-experimental design. Likewise, a sample made up of 1057 costaleros from the Spanish cities of Almería, Cádiz, Córdoba, Granada, Huelva, Jaén, Málaga, and Sevilla, corresponding to 930 males and 127 females (M = 31.26 ± S.D. = 7.60). Convenience sampling was used to recruit the participants.

### 2.2. Instruments

Accelerometers. One ActiGraph GT3Xw (ActiGraph^TM^, LLC, Pensacola, FL, USA) accelerometer was used for each costalero. Data collection was done through the triaxial function for every 10 s. Cut-off points were adjusted for adults [17] to categorize sedentary physical activity intensity (SPA) < 99 counts per minute (cpm), 100–1951 cpm for light physical activity (LPA), 1952–5724 cpm for moderate physical activity (MPA), and >5725 cpm for vigorous physical activity (VPA). MVPA, METs, Kcal, and steps were also obtained. Before the start of each practice session, the researchers placed the accelerometers just above the right hip of the costaleros, under their clothes, and collected each one at the end.

Health-related quality of life. The SF-36 questionnaire measures the dimensions that make up the quality of life in adult populations [18] using the Spanish version of Alonso, Prieto and Anto [19] with a 0.87 Crombach alpha. The eight dimensions that make up the scale are calculated by means of the average of the sum of the questions that make up each of the constructs. These concepts are: (a) physical function (PF), (b) role physical (RF), (c) bodily pain (BP), (d) general health (GH), (e) vitality (VT), (f) social functioning (SF), (g) role emotional (RE), and (h) mental health (MH). In addition to the eight health concepts, the SF-36 includes the general concept of changes in the perception of the current health status and that of the previous year. The SF-36 is a self-administered instrument, containing 36 questions. For each scale, the answers to each question are coded and recoded (10 questions), and the results are transferred to a scale from 0 (worst health) to 100 (best health) (See Supplementary Materials).

Socio-demographic questions relating to sex, age, city, and if they had suffered any injury were also added.

### 2.3. Procedure

The first step was to request permission from the University of Granada to carry out the research, obtaining ethics committee 641/CEIH/2019. The study was carried out between the months of March and April 2019. The participants were instructed by the researchers to fill out the questionnaire correctly, ensuring their total anonymity. The test was sent by email using the Google forms application to the 1057 participants that constitute the sample. Once the test was completed, 40 volunteers from the entire sample were asked to carry the accelerometer during the 8 h of the procession. Anonymity and confidentiality of the data were ensured. Data were collected and their quality was confirmed, whilst ensuring throughout that the process conformed to the ethical principles for research defined in the Helsinki´s Declaration in 1975 and subsequently updated in Brazil in 2013.

### 2.4. Statistical Analysis

Actilife 6.7.1 software was used to obtain the data collected by accelerometers (ActiGraph^TM^, LLC, Pensacola, FL, USA). Subsequently, the data were analysed using the statistic package SPSS^®^ 25.0 (IBM, Chicago, IL, USA). Descriptive, inferential, and correlative analyses were performed to calculate means, 95% confidence intervals, the standard error of estimation, and the coefficient of variation. Pairwise comparisons were used to assess differences between sex and age groups. Several unidirectional analyses of variance (ANOVA) were also performed to assess differences between age ranges of participants in all variables like injury, age, sex, and the eight dimensions of SF-36 test. Therefore, Pearson’s bivariate correlations were also calculated between all the above-mentioned variables that were being studied to assess the relationships between them.

## 3. Results

Results obtained in the present investigation show that 87.98% (*n* = 930) were men, while 12.02% (*n* = 127) were women. Moreover, about 70% of men were between 18 and 35 years old as well as women; 30% of them were between 36 and 61 years old. Regarding the variable having suffered some injury, 54.1% (*n* = 572) claimed to have suffered some type of injury, while 45.9% (*n* = 485) had not suffered any.

Table 1 shows the data relating to sex and age from the sample depending on the intensity of the physical activity carried out during the procession. For SPA, the average values are higher in all age ranges for men, finding statistically significant differences (*p* < 0.05) for the 18–61 year age range. For LPA the average values are higher, although no statistically significant differences were found. The highest average values are found in the MPA intensity, although no statistically significant differences were found either for sex or age. Finally, for the VPA intensity, statistically significant differences (*p* < 0.05) were found in all age groups. In all of them, men had higher averages than women. Men between 18 and 35 years old are those who present the highest average in terms of VPA, with statistically significant differences (*p* < 0.05) with respect to the 36 to 61 year-old range.

Table 2 shows the different dimensions of quality of life in relation to the costaleros’ age groups. There were no statistically significant differences in the general health, social functioning, vitality, mental health, and role emotional dimensions. However, statistical differences were found in favour of participants aged 18 to 35 years for the dimensions bodily pain (*f* = 1.87; *p* = 0.020), role physical (*f* = 6.46; *p* = 0.000), and physical functioning (*f* = 0.90; *p* = 0.031).

Likewise, Table 3 shows the bivariate correlations between quality of life, the type of physical activity performed during the procession, and whether or not one has suffered an injury. The data obtained show how there is a strong correlation between suffering an injury and the other variables. Similarly, the strongest correlation is found between having suffered an injury with bodily pain (*r* = 0.866) and negative with general health (*r* = −0.817). There is also strong negative correlation between having suffered an injury with vitality (*r* = −0.765), MPA (*r* = −0.714), and VPA (*r* = −0.756), and moderate correlation with mental health (*r* = 0.681), role physical (*r* = −0.598), and physical functioning (*r* = −0.532). There is also strong and negative correlation between VPA and bodily pain (*r* = −0.784). On the other hand, moderate correlations were also found between VPA and general health (*r* = 0.685) and between MPA, general health (*r* = 0.612), and bodily pain (*r* = 0.532).

## 4. Discussion

Due to the lack of studies where the population are costaleros, it has been deemed suitable to establish comparisons with studies whose samples are close to the sample characteristics in this study. Therefore, the adult population as a whole has been considered as suitable to establish such comparisons. In view of the characteristics adhered to costaleros, that is, they do not have to practice physical activity regularly and must be at least 18 years old, the inclusion of this type of study is justified. Likewise, the main objective of this research is to determine the intensity of physical activity that costaleros carry out during a Holy Week procession.

### 4.1. Physical Activity Intensity

Many studies use accelerometers to determine with better accuracy the intensity of physical activity in daily tasks or tasks that are not socially considered as exercise [20,21]. The data obtained from the accelerometers show how the intensity of the effort made is focused on moderate physical activity in most parts of the procession. There are also periods of light and vigorous physical activity, although in smaller proportions. Likewise, it must be pointed out that the time of sedentary physical activity corresponds to the periods in which the 40 volunteers costaleros is resting. In turn, the data show that men are more active than women [22,23,24]. These data differ from the study carried out on English adults (40–69 years of age) [25] in which it is noted that women are less sedentary than men.

On the other hand, it is noteworthy how the costaleros are alternating their different intensities of physical activity during the course of the procession, regardless of sex or age. Regarding the differences between age groups, the sample between 18 and 35 years old finds great differences in relation to the participants who are between 36 and 61 years old. These differences are based on the age differences between the two groups and, consequently, on physical ability. It is important to highlight the difference between sports such as powerlifting [26,27,28,29], where the sack-lifters perform the lift assisted by a small explosive jump and followed by movement for about 10 min.

Likewise, the study by Chapman et al. [30], in which the different loads performed in a parallel squat with support on C7 vertebra are studied, shows how athletes need to perform 2/3 repetitions in order to induce maximum muscle activation and avoid injuries. In contrast, it happens with costaleros, since they do not perform any activation prior to the lifting activity that can be repeated about 20/30 times per procession. On the other hand, it is also shown how it can be compared to resistance training [31] of parallel squats.

### 4.2. Quality of Life

Taking into account the age range, it is possible to appreciate the findings regarding the costaleros’ perceived quality of life. The results show how the youngest population of 18–35 years old exhibit a better physical role and correct body functioning against the population between 36–61 years old. Likewise, it can be concluded that the older population has higher levels of some bodily disease.

The results show how the studied population presents high values in the dimensions of quality of life in relation to the practice of physical activity [32,33,34,35]. Due to the fact that it is a group that includes a wide age range, the differences in terms of quality-of-life dimensions in relation to the proposed age ranges can be seen. Accordingly, there are several studies that present similar data with the adult population [36,37,38]. In addition, the study by Blom et al. [36] conducted in a population of 524 adults found benefits on all eight dimensions after an intervention using a three-month physical activity program. On the other hand, Wyke et al. [37] found benefits in 1113 adults who were doing LPA and MPA. Generally, the costaleros present a good quality of life related to the practice of physical activity that they carry out during Easter Week. Similar data were obtained in a study on the effects of Pilates practice among adults [39].

The dimension with the lowest scores is body pain. Indeed, it is evident that subjects cannot present significant physical limitations to practice this type of physical activity because they could not play their role properly if they were not. Likewise, the study by Robles-Romero et al. [6] carried out with costaleros where the BMI of the costaleros is measured in relation to blood pressure was found that the group of costaleros is exposed to high physical demands with little previous physical preparation.

In addition, social factors such as friends help participants to engage themselves in this type of physical activity, even though they may have some previous physical ailment or may have suffered an injury [40].

By relating both variables, positive relationships are observed between quality of life and moderate (MPA), vigorous (VPA), and light (LPA) physical activity. Different intensities of PA were significantly associated with different dimensions of quality of life [41]. Likewise, it is also found there is strong positive correlation between having suffered an injury with SPA [38]. In contrast, negative differences were found between being injured and MPA and VPA intensities [42,43].

One of the strengths of the present study is the large sample size (1057 participants) in relation to the characteristics of the studied population. We consider this sample to be more than representative for a study involving costaleros. In addition, the use of accelerometers with 40 volunteers has made it possible to verify the type and intensity of physical activity practiced by the costaleros during a procession more accurately. Perhaps, one of the possible limitations is the measurement using the accelerometers that, to check the routine activity that each individual performs, they should have worn the accelerometer during the whole day. In addition, the transversal design of the study limits somewhat the causal direction of the associations between quality of life, injuries, and PA, since not only the physical or mental health of an individual could have a direct impact on the intensity of physical activity. Another limitation is the impossibility of contrasting our findings with other research carried out among costaleros, since at present, there are only three empirical investigations related to health status and physical activity practice in the population studied.

## 5. Conclusions

The present study examined the relationships between quality of life and the intensity of physical activity practiced by costaleros. In addition, we analysed the relationships of these variables with sex, age, and having suffered some type of injury. The findings suggest that costaleros have mainly a moderate intensity of physical activity during a procession. In addition, it has been found that the intensity of physical activity is higher for males than for females. In relation to age, it has been found that males and females of 18 and 35 years of age have the highest average in terms of bodily pain, role physical, and physical functioning with respect to the 36 to 61 year-old range, regarding the issues included in the quality-of-life assessment. It should be noted that no statistically significant differences were observed in relation to general health, mental health, or social functioning, which may indicate that being costalero is an inclusive physical activity that is positively related to health.

In addition, it can be concluded that costaleros suffer few injuries inversely related to the intensity of physical activity performed. It is important to establish, as future strategies, an intervention program whereby training or stretching guidelines are taught prior to the performance of the costalero’s physical activity. It would also be desirable to include anthropometric data, such as height, weight, obesity, or diet quality to establish a better profile of the costalero.

## Figures and Tables

**Table 1:** Averages of physical activity measured with accelerometer by age and sex during the procession.

Years Sex	Sample Size	Sedentary PA			Light PA			Moderate PA			Vigorous PA		
			95% C.I.			95% C.I.			95% C.I.			95% C.I.	
		M	from	to	M	from	to	M	from	to	M	from	to
18–61	1057	10.8	8.6	12.1	15.7	13.1	17.4	31.3	29.7	33.5	5.8	3.6	7.4
Males	930	11.2 *	8.9	13.6	15.6	13.2	17.1	31.1	29.3	33.1	6.4 *	4.2	8.8
Females	127	9.7	7.5	11.3	15.1	12.7	17.6	29.7	27.4	31.6	5.2	3.1	7.4
18–35	744	8.8	6.5	10.3	16.3	14.1	18.2	20.8	18.3	22.8	8.6 *	6.6	10.9
Males	654	9.1	7.3	11.2	16.5	14.6	19.1	29.5 *	26.6	31.4	8.8	6.9	11.1
Females	90	8.4	6.7	10.7	16.1	13.9	18.7	21.2	19.8	23.4	8.4	6.7	10.5
36–61	313	9.1	7.4	11.5	15.2	13.7	17.8	19.3	17.4	21.8	4.1 *	2.8	6.2
Males	276	9.8	7.1	11.8	14.9	12.5	16.4	19.9	17.2	22.1	4.3	3.1	6.4
Females	27	8.2	6.1	10.3	15.4	13.2	17.3	18.2	16.5	20.3	3.9	1.8	5.2

* Statistically significant differences at level *p* < 0.05. PA: Physical Activity.

**Table 2:** Averages between quality-of-life dimensions and sample age.

Variable	18–61		18–35		36–61		Levene Test		Sig. (Bilateral)	ES (d)	95% CI
	M	S.D.	M	S.D.	M	S.D.	F	Sig.			
GH	14.88	1.79	14.83	1.77	14.98	1.81	1.52	0.217	0.061	0.055	[14.70; 14.98]
SF	6.92	0.90	6.93	0.90	6.91	0.90	0.12	0.725	0.790	0.028	[6.81; 7.01]
V	13.71	2.03	13.78	2.02	13.55	2.06	2.79	0.005	0.173	0.063	[13.32; 13.92]
MH	19.52	1.89	19.50	1.91	19.57	1.84	0.26	0.605	0.251	0.058	[19.36; 19.77]
BP	3.83	1.97	3.70	2.01	3.88	1.87	1.83	0.176	0.020 *	0.061	[3.49; 4.03]
RE	5.60	0.89	5.59	0.90	5.63	0.89	0.44	0.507	0.353	0.028	[5.54; 5.73]
RP	7.63	1.03	7.75	1.10	7.15	0.83	6.46	0.011	0.000 *	0.032	[7.49; 7.84]
PF	29.62	1.02	29.67	1.10	29.60	0.81	0.90	0.042	0.031 *	0.032	[29.52; 29.76]

Note: GH: general health; SF: social functioning; V: vitality; MH: mental health; BP: bodily pain; RE: role emotional; RP: role physical; PF: physical functioning; * *p* < 0.05.

**Table 3:** Bivariate correlations among variables.

	GH	SF	V	MH	BP	RE	RP	PF	Injury	SPA	LPA	MPA	VPA
GH	-	0.014	0.166 **	0.103 **	0.052	−0.066 *	−0.065 *	−0.151 **	−0.817 **	0.023	−0.237 **	0.612 **	0.685 **
SF		-	0.109 **	0.055	0.030	0.033	−0.057	−0.028	−0.058	0.021	0.142 **	0.213 **	0.498 **
V			-	0.104 **	−0.013	−0.129 **	−0.104 **	−0.071*	−0.765 **	0.251 **	0.102 **	0.437 **	0.367 **
MH				-	−0.160 **	0.338 **	0.145 **	0.115 **	0.681 **	0.054	0.098 *	0.269 **	0.235 **
BP					-	−0.265 **	−0.661 **	−0.346 **	0.866 **	0.354 **	0.471 **	0.532 **	−0.784 **
RE						-	0.303 **	0.263 **	−0.042	0.036	0.365 **	0.258 **	0.261 **
RP							-	0.475 **	−0.598 **	0.050	0.089 *	0.217 **	0.116 **
PF								-	−0.532 **	0.487 **	0.545 **	0.463 **	0.129 **
Injury									-	0.647 **	0.416 **	−0.714 **	−0.756 **
SPA										-	0.124 **	0.287 **	0.251 **
LPA											-	0.314 **	0.047
MPA												-	0.259 **
VPA													-

Note: GH: general health; SF: social functioning; V: vitality; MH: mental health; BP: bodily pain; RE: role emotional; RP: role physical; PF: physical functioning; SPA: sedentary physical activity; LPA: light physical activity; MPA: moderate physical activity; VPA: vigorous physical activity. ** *p* < 0.01 * *p* < 0.05.

## Supplementary Materials

The following are available online at https://www.mdpi.com/1424-8220/20/19/5641/s1.
