# Supplementary material for: Spanish Costaleros’ Physical Activity and Their Quality of Life
Source: Sensors (Basel). 2020 Oct 2;20(19):5641. doi: 10.3390/s20195641 (PMC7582451; doi:10.3390/s20195641)
Supplement: Supplementary file 1 [file sensors-20-05641-s001.pdf]

sensors

Supplementary

Spanish Costaleros' Physical Activity and Their Quality of Life

José Luis Ubago-Jiménez, Félix Zurita-Ortega, Pilar Puertas-Molero * and Gabriel González-Valero

Department of Didactics of Musical, Plastic and Corporal Expression, University of Granada, Granada 18071, Spain; jlubago@ugr.es (J.L.U.-J.); felixzo@ugr.es (F.Z.-O.); ggvalero@ugr.es (G.G.-V.)

* Correspondence: pilarpuertas@correo.ugr.es; Tel.: +34-958-246-685

Spanish version of the SF-36 Health Survey

1. En general, usted diría que su salud es:

1 Excelente 2 Muy Buena 3 Buena 4 Regular 5 Mala

2. ¿Cómo diría que es su salud actual, comparada con la de hace un año?

1 Mucho mejor ahora que hace un año

2 Algo mejor ahora que hace un año

3 Más o menos igual que hace un año

4 Algo peor ahora que hace un año

5 Mucho peor ahora que hace un año

3. Su salud actual, ¿le limita para hacer esfuerzos intensos, tales como correr, levantar objetos pesados, o participar en deportes agotadores?

1 Sí, me limita mucho 2 Sí, me limita un poco 3 No, no me limita nada

4. Su salud actual, ¿le limita para hacer esfuerzos moderados, como mover una mesa, pasar la aspiradora, jugar a los bolos o caminar más de una hora?

1 Sí, me limita mucho 2 Sí, me limita un poco 3 No, no me limita nada

5. Su salud actual, ¿le limita para coger o llevar la bolsa de la compra?

1 Sí, me limita mucho 2 Sí, me limita un poco 3 No, no me limita nada

6. Su salud actual, ¿le limita para subir varios pisos por la escalera?

1 Sí, me limita mucho 2 Sí, me limita un poco 3 No, no me limita nada

7. Su salud actual, ¿le limita para subir un solo piso por la escalera?

1 Sí, me limita mucho 2 Sí, me limita un poco 3 No, no me limita nada

8. Su salud actual, ¿le limita para agacharse o arrodillarse?

1 Sí, me limita mucho 2 Sí, me limita un poco 3 No, no me limita nada

9. Su salud actual, ¿le limita para caminar un kilómetro o más?

1 Sí, me limita mucho 2 Sí, me limita un poco 3 No, no me limita nada

10. Su salud actual, ¿le limita para caminar varias manzanas (varios centenares de metros)?

1 Sí, me limita mucho 2 Sí, me limita un poco 3 No, no me limita nada

11. Su salud actual, ¿le limita para caminar una sola manzana (unos 100 metros)?

1 Sí, me limita mucho 2 Sí, me limita un poco 3 No, no me limita nada

12. Su salud actual, ¿le limita para bañarse o vestirse por sí mismo?

1 Sí, me limita mucho 2 Sí, me limita un poco 3 No, no me limita nada

13. Durante las 4 últimas semanas, ¿tuvo que reducir el tiempo dedicado al trabajo o a sus actividades cotidianas, a causa de su salud física?

1 Sí 2 No

14. Durante las 4 últimas semanas, ¿hizo menos de lo que hubiera querido hacer, a causa de su salud física?

1 Sí 2 No

15. Durante las 4 últimas semanas, ¿tuvo que dejar de hacer algunas tareas en su trabajo o en sus actividades cotidianas, a causa de su salud física?

1 Sí 2 No

16. Durante las 4 últimas semanas, ¿tuvo dificultad para hacer su trabajo o sus actividades cotidianas (por ejemplo, le costó más de lo normal), a causa de su salud física?

1 Sí 2 No

17. Durante las 4 últimas semanas, ¿tuvo que reducir el tiempo dedicado al trabajo o a sus actividades cotidianas, a causa de algún problema emocional (como estar triste, deprimido, o nervioso?

1 Sí 2 No

18. Durante las 4 últimas semanas, ¿hizo menos de lo que hubiera querido hacer, a causa de algún problema emocional (como estar triste, deprimido, o nervioso)?

1 Sí 2 No

19. Durante las 4 últimas semanas, ¿no hizo su trabajo o sus actividades cotidianas tan cuidadosamente como de costumbre, a causa de algún problema emocional (como estar triste, deprimido, o nervioso)?

1 Sí 2 No

20. Durante las 4 últimas semanas, ¿hasta qué punto su salud física o los problemas emocionales han dificultado sus actividades sociales habituales con la familia, los amigos, los vecinos u otras personas?

1 Nada 2 Un poco 3 Regular 4 Bastante 5 Mucho

21. ¿Tuvo dolor en alguna parte del cuerpo durante las 4 últimas semanas?

1 No, ninguno 2 Sí, muy poco 3 Sí, un poco 4 Sí, moderado 5 Sí, mucho 6 Sí, muchísimo

22. Durante las 4 últimas semanas, ¿hasta qué punto el dolor le ha dificultado su trabajo habitual (incluido el trabajo fuera de casa y las tareas domésticas)?

1 Nada 2 Un poco 3 Regular 4 Bastante 5 Mucho

23. Durante las 4 últimas semanas, ¿cuánto tiempo se sintió lleno de vitalidad?

1 Siempre 2 Casi siempre 3 Muchas veces 4 Algunas veces 5 Sólo alguna vez 6 Nunca

24. Durante las 4 últimas semanas, ¿cuánto tiempo estuvo muy nervioso?

1 Siempre 2 Casi siempre 3 Muchas veces 4 Algunas veces 5 Sólo alguna vez 6 Nunca

25. Durante las 4 últimas semanas, ¿cuánto tiempo se sintió tan bajo de moral que nada podía animarle?

1 Siempre 2 Casi siempre 3 Muchas veces 4 Algunas veces 5 Sólo alguna vez 6 Nunca

26. Durante las 4 últimas semanas, ¿cuánto tiempo se sintió calmado y tranquilo?

1 Siempre 2 Casi siempre 3 Muchas veces 4 Algunas veces 5 Sólo alguna vez 6 Nunca

27. Durante las 4 últimas semanas, ¿cuánto tiempo tuvo mucha energía?

1 Siempre 2 Casi siempre 3 Muchas veces 4 Algunas veces 5 Sólo alguna vez 6 Nunca

28. Durante las 4 últimas semanas, ¿cuánto tiempo se sintió desanimado y triste?

1 Siempre 2 Casi siempre 3 Muchas veces 4 Algunas veces 5 Sólo alguna vez 6 Nunca

29. Durante las 4 últimas semanas, ¿ cuánto tiempo se sintió agotado?

1 Siempre 2 Casi siempre 3 Muchas veces 4 Algunas veces 5 Sólo alguna vez 6 Nunca

30. Durante las 4 últimas semanas, ¿ cuánto tiempo se sintió feliz?

1 Siempre 2 Casi siempre 3 Muchas veces 4 Algunas veces 5 Sólo alguna vez 6 Nunca

31. Durante las 4 últimas semanas, ¿cuánto tiempo se sintió cansado?

1 Siempre 2 Casi siempre 3 Muchas veces 4 Algunas veces 5 Sólo alguna vez 6 Nunca

32. Durante las 4 últimas semanas, ¿con qué frecuencia la salud física o los problemas emocionales le han dificultado sus actividades sociales (como visitar a los amigos o familiares)?

1 Siempre 2 Casi siempre 3 Muchas veces 4 Algunas veces 5 Sólo alguna vez 6 Nunca

33. Creo que me pongo enfermo más fácilmente que otras personas.

1 Totalmente cierta 2 Bastante cierta 3 No lo sé 4 Bastante falsa 5 Totalmente falsa

34. Estoy tan sano como cualquiera.

1 Totalmente cierta 2 Bastante cierta 3 No lo sé 4 Bastante falsa 5 Totalmente falsa

35. Creo que mi salud va a empeorar.

1 Totalmente cierta 2 Bastante cierta 3 No lo sé 4 Bastante falsa 5 Totalmente falsa

36. Mi salud es excelente.

1 Totalmente cierta 2 Bastante cierta 3 No lo sé 4 Bastante falsa 5 Totalmente falsa
